# Myocardial stunning: mechanisms, molecular insights, and gaps in knowledge

**DOI:** 10.1042/BSR20253410

**Published:** 2025-12-16

**Authors:** Amin Al-Awar, Abdul Waheed Khan, Shafaat Hussain

**Affiliations:** 1 Department of Molecular and Human Genetics, Baylor College of Medicine, Houston, Texas, U.S.A.; 2 Department of Diabetes, School of Translational Medicine, Monash University, Melbourne, Australia; 3 Department of Molecular and Clinical Medicine, Institute of Medicine, Gothenburg University, Gothenburg, 41345, Sweden

**Keywords:** epigenetics, myocardial stunning, phospho-proteomics, proteomics, ROS

## Abstract

Myocardial stunning, characterized by transient post-ischemic contractile dysfunction despite the restoration of coronary blood flow, has been a pivotal subject of cardiovascular research. Initially perceived as a consequence of irreversible myocardial damage and fibrosis, the concept evolved in the 1970s when studies revealed that reperfusion could salvage ischemic myocardium, leading to therapies like thrombolysis, percutaneous coronary intervention, and coronary artery bypass grafting. The phenomenon of myocardial stunning was first detailed by Heyndrickx et al. and later termed by Braunwald and Kloner, challenging previous views by demonstrating that reperfusion can cause temporary yet reversible dysfunction without necrosis. Extensive research elucidated mechanisms involving reactive oxygen species (ROS), calcium overload, and impaired excitation–contraction coupling. Recent advances in proteomics and phospho-proteomics identified molecular changes linked to contractile dysfunction, extracellular matrix damage, and apoptosis. The role of epigenetics has also garnered attention for its potential to influence myocardial stunning and offer therapeutic avenues. This review comprehensively explores the historical and mechanistic landscape of myocardial stunning, recent molecular insights, and its clinical relevance. Future research directions emphasize advanced proteomic and phosphor-proteomic analyses, epigenetic mechanisms, clinical translation, non-invasive diagnostics, ROS role clarification, ischemia preconditioning impacts, and integrative systems biology. Addressing these areas will enhance our understanding and lead to improved therapeutic strategies for ischemic heart disease.

## Introduction

Myocardial stunning is a condition characterized by transient post-ischemic contractile dysfunction despite the restoration of normal coronary blood flow [1]. Indeed**,** myocardial stunning is a central feature of acute ischemia/reperfusion injury, representing a reversible contractile deficit that manifests specifically upon the restoration of coronary flow. It occupies a critical position on the pathophysiological spectrum of ischemic heart disease, which ranges from fully reversible dysfunction to irreversible infarction. The duration of the ischemic insult is the primary determinant of this outcome; while prolonged ischemia leads to necrosis, even a brief episode is sufficient to cause stunning, a state of viable myocardium that has been ‘salvaged but not saved’ from functional impairment. This distinction is translationally critical. It underscores that successful reperfusion, the cornerstone of modern therapy for acute myocardial infarction (MI), does not guarantee immediate functional recovery. Therefore, understanding and mitigating myocardial stunning is essential for improving post-ischemic outcomes, moving beyond mere myocardial salvage to achieving true functional salvage [2,3].

Initially, it was thought to result from irreversible myocardial damage and fibrotic replacement [4]. However, our understanding evolved significantly in the 1970s when studies demonstrated that reperfusion could salvage ischemic myocardium [5], leading to the development of therapies such as thrombolysis, percutaneous coronary intervention (PCI) [4], and coronary artery bypass graft (CABG) surgery [4].

Heyndrickx et al. were the first to detail myocardial stunning, revealing that reperfusion can cause temporary, reversible contractile dysfunction, challenging the view that myocardial function would promptly or permanently recover post-reperfusion [6]. Braunwald and Kloner further solidified the concept by coining the term ‘myocardial stunning’ to describe the delayed recovery of myocardial function, even after the complete restoration of blood supply and in the absence of necrosis [7,8].

Extensive research work investigated the mechanisms of myocardial stunning, focusing on the roles of reactive oxygen species (ROS) [9,10], calcium overload [11–13], and impaired excitation–contraction coupling [1]. These mechanisms are not mutually exclusive and often interact to influence myocardial function. Recent advances in unbiased proteomics and phospho-proteomics have provided deeper insights into the molecular alterations associated with myocardial stunning, identifying significant changes in protein expression and phosphorylation patterns linked to contractile dysfunction, extracellular matrix (ECM) damage, and apoptotic cell death [14,15].

In addition to these molecular insights, the potential role of epigenetic regulation in myocardial stunning has gained attention [16,17]. Epigenetic modifications, including DNA methylation, histone modifications, and non-coding RNA (ncRNA) interactions, have been shown to influence gene expression without altering the DNA sequence [18,19]. These modifications can play a crucial role in regulating responses to ischemia-reperfusion (I/R) injury [20–22], potentially impacting the development and resolution of myocardial stunning. Understanding the epigenetic landscape in myocardial stunning could offer new therapeutic avenues for mitigating its effects and improving cardiac recovery. In conclusion, myocardial stunning represents a complex interplay of ischemic injury and reperfusion dynamics, with significant implications for patient care. Continued research into the molecular and epigenetic mechanisms underlying this condition, coupled with advancements in therapeutic strategies, has the potential to transform the management of myocardial stunning and improve cardiac health outcomes.

This review aims to comprehensively explore the landscape of myocardial stunning, from its historical definitions and mechanistic theories to recent advancements in proteomics, phospho-proteomics, and epigenetics. It also examines the clinical relevance of myocardial stunning, highlighting its implications in various clinical settings and its potential as a target for therapeutic intervention. Through this detailed exploration, we aim to enhance the understanding and guide future research and clinical strategies to mitigate the impact of myocardial stunning on cardiac health.

## Defining myocardial stunning: a contemporary overview

Classically, contractile dysfunction was viewed as a loss of viable myocardium and its replacement with fibrotic tissues in patients with coronary artery disease (CAD) [4]. In 1972, Maroko et al. demonstrated for the first time in an experimental dog model of MI that 3 hours of coronary reperfusion results in the salvage of myocardium tissue [5]. Shortly after, in the seventies, the reperfusion or revascularization in patients with MI became feasible as technologies such as thrombolysis, PCI [4], and CABG surgery were discovered. The feasibility and benefits of reperfusion therapy in patients with MI were apparent in terms of myocardial salvage and symptom relief [23–25]. It was believed that the myocardial function would recover almost promptly upon reperfusion or would not recover completely when the myocardium had been exposed to more prolonged ischemia and infarction. However, this view was challenged when the phenomenon of myocardial stunning was discovered by Heyndrickx et al. because reperfusion not only reverts the myocardial ischemia but also results in pathophysiological alterations.

Similarly, myocardial ischemia not only induces deleterious changes but also initiates mechanisms protective in nature. Heyndrickx et al. demonstrated in awake dogs that after 5 to 15 minutes of coronary occlusion, the recovery of contractile function was delayed to 24 hours, whereas the electrocardiogram normalized more or less immediately [6]. During that time, there was some skepticism regarding the concept that the myocardium function would not recover instantly after reperfusion, illustrated by the rejection of a manuscript in which the observations that the contractile function did not revert entirely to normal in 30 minutes in an experimental dog model of MI was characterized as a possible artifact [26]. In 1982, Braunwald and Kloner reported the delayed recovery of myocardial contractile function in the absence of necrosis and eventually coined the term ‘myocardial stunning’ for this phenomenon [7]. Therefore, this study by Heyndrickx et al. laid a solid foundation for understanding the mechanisms of myocardial stunning, which has contributed significantly to our current knowledge in this area of cardiac research.

## Perfusion–contraction mismatch: a defining feature of myocardial stunning

A defining physiological feature of myocardial stunning is the uncoupling between myocardial perfusion and contractile function during reperfusion, a phenomenon known as perfusion–contraction mismatch. Under normal physiological and ischemic conditions, myocardial blood flow and function are typically matched; when perfusion decreases, contractile function declines proportionally. This quantitative relationship was first established by Vatner, who demonstrated a close exponential correlation between reductions in regional myocardial blood flow and segmental shortening during graded ischemia in conscious dogs [27]. Subsequent work by Gallagher and colleagues extended these findings to exercise-induced ischemia, showing that the relationship between myocardial blood flow and systolic wall thickening remains proportional under physiological stress, confirming the principle of perfusion–contraction matching [28].

However, myocardial stunning breaks this coupling. After brief ischemia and timely reperfusion, regional blood flow is restored, yet contractile dysfunction persists, sometimes for hours or days. Heyndrickx et al. observed persistent wall motion abnormalities despite fully restored perfusion in post-ischemic myocardium [6]. Later, Ambrosio et al. provided experimental evidence that ROS generated during early reperfusion impair myocardial function independently of blood flow, implicating oxidative stress as a major mechanism [29]. Kusuoka et al. and Gao et al. demonstrated that impaired calcium cycling and reduced myofilament calcium responsiveness underlie the transient loss of contractility [30,31]. Bolli’s 1990 review integrated these findings into a unified mechanistic framework, emphasizing oxidative stress, calcium overload, and excitation–contraction uncoupling as key determinants of stunning [32].

The physiological implications of these mechanisms are best understood in the context of the regional flow–function relationship. Analyses of this relationship have demonstrated that, in stunned myocardium, blood flow is fully restored, whereas contractile performance remains impaired, establishing a clear perfusion–contraction mismatch [33]. This dissociation cannot be explained by oxygen supply–demand imbalance, as myocardial perfusion in stunned regions consistently exceeds the critical threshold (~8–10 μl·g⁻¹·beat⁻¹) required for oxidative metabolism [33]. Instead, the mismatch reflects reperfusion-related cellular disturbances, such as oxidative stress, calcium overload, and impaired excitation–contraction coupling that transiently prevent the translation of adequate perfusion into normal mechanical function. Consequently, myocardial stunning should be recognized as a reperfusion phenomenon, distinct from persistent ischemia [33].

Within the continuum of ischemia–reperfusion syndromes, the flow–function relationship defines a hierarchy of myocardial states distinguished by the degree of coupling between perfusion and contraction. Normal myocardium exhibits matched flow and function, hibernating myocardium shows proportional down-regulation of both, and stunned myocardium uniquely demonstrates restored perfusion with sustained contractile depression [34]. This conceptual framework clarifies that perfusion–contraction mismatch is the physiological signature of myocardial stunning, distinguishing it from other ischemic adaptations [33,34].

## Mechanisms of myocardial stunning

Several theories were proposed in the 1980s regarding the mechanisms of myocardial stunning, such as the generation of oxygen-derived free radicals, calcium overload, decreased responsiveness to myofilaments, excitation–contraction uncoupling due to sarcoplasmic reticulum (SR) dysfunction, insufficient energy production by mitochondria, impaired energy use by myofibrils, impairment of sympathetic neural responsiveness, impairment of myocardial perfusion, damage of the extracellular collagen matrix, and impaired excitation. Bolli and Marbán have thoroughly reviewed these theories [32,35], and since their comprehensive discussion, there have been few novel developments in this area. Among them, the most appreciated mechanisms implicated in myocardial stunning are the increased formation of ROS that results in oxidative modification of the contractile apparatus and alterations in excitation–contraction coupling. These two mechanisms are not mutually exclusive and interact with each other.

### The role of ROS in myocardial stunning

The notion that ROS plays a significant role in the pathophysiology of myocardial stunning goes back to the 1980s when several investigators postulated that myocardial stunning is partly a consequence of ROS [35,36]. Myers et al. for the first time tested this idea in open-chest dogs in which the left anterior descending coronary artery was occluded for 15 minutes, followed by 2 hours of reperfusion. Administration of oxygen-free radical scavengers superoxide dismutase (SOD) and catalase significantly enhances the recovery of function in this model [37]. Later, other laboratories produced similar results using SOD and catalase in similar experimental dog, canine, and rabbit models [10,38,39]. Furthermore, it was demonstrated that potent and cell-permeable antioxidant mercaptopropionyl glycine could markedly attenuate myocardial stunning implemented just before the restoration of flow, even in the absence of any treatment during ischemia, proposing that myocardial stunning is, at least in part, a form of ROS-mediated phenomenon [9]. Similarly, antioxidants such as dimethyl thiourea, desferrioxamine, and others were also found promising to attenuate myocardial stunning after 15 minutes of ischemia in different experimental animal models [40–42]. All these studies provided an impressive body of evidence supporting the implication of ROS in myocardial stunning but were limited by the fact that this evidence was indirect. Therefore, it was imperative to directly measure the generation of free radicals in the presence and absence of antioxidants to validate the ROS hypothesis in myocardial stunning. Thus, using electron paramagnetic resonance spectroscopy and the spin trap, α-phenyl N-tert-butyl nitrone, ROS was directly demonstrated in open-chest dogs with 15 minutes of coronary artery occlusion and subsequent reperfusion [43]. This study demonstrated that ROS generation begins within a few minutes of coronary occlusion, is very significant in the early phase of reperfusion, decreases in later stages of reperfusion, but does not cease and persists until 3 hours of reperfusion [43]. Furthermore, ROS production was inversely proportional to the magnitude of ischemic flow reduction [43], in analogy to a previously documented inverse relationship between ischemic flow and degree of post-ischemic contractile dysfunction [44]. However, similar findings were observed in conscious dogs subjected to a 15 minutes coronary occlusion, indicating that such findings are well applicable in more physiological conditions, and ROS is necessary for developing myocardial stunning [35].

**Figure 1:**
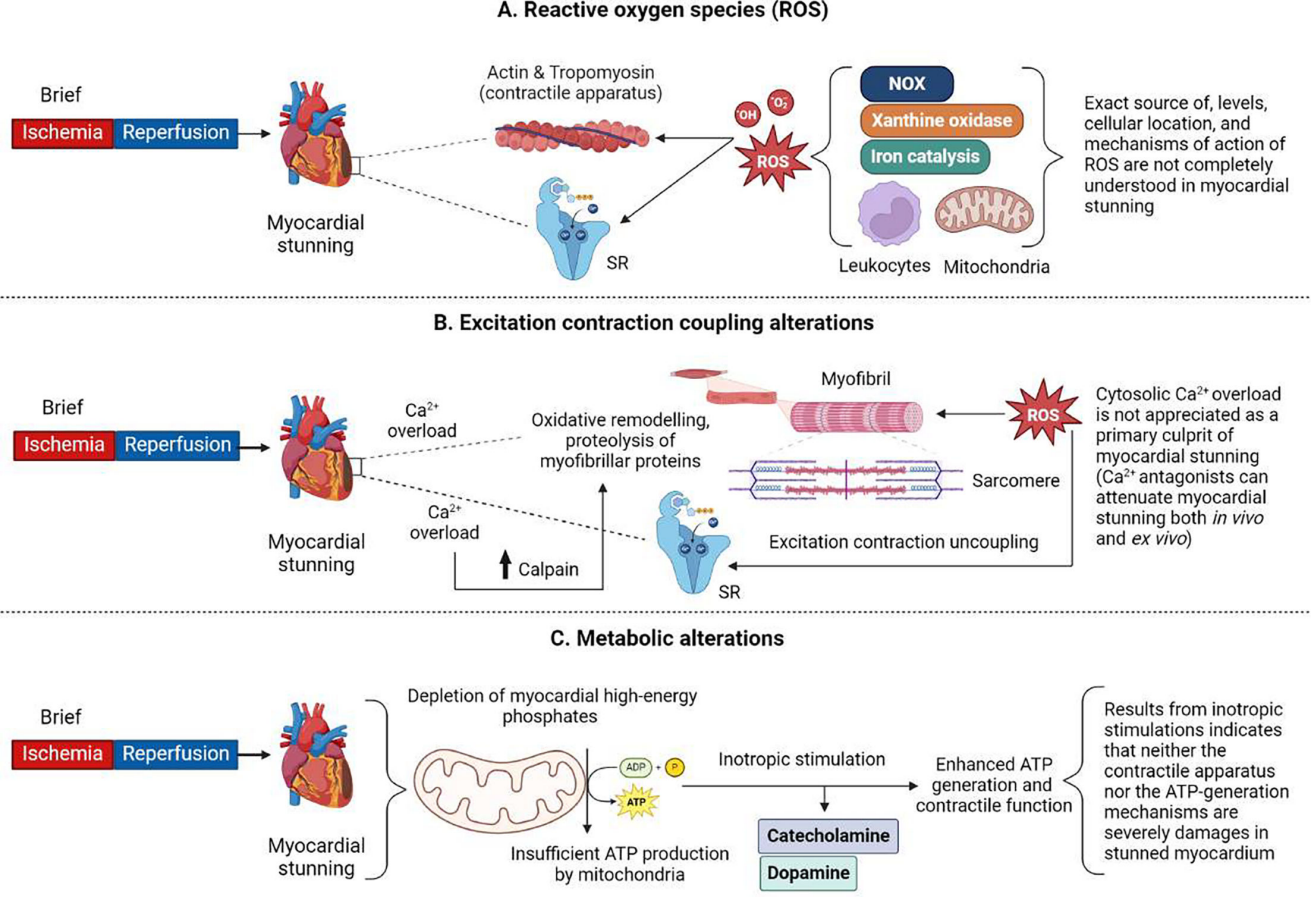
Classical mechanisms underlying myocardial stunning. (**A**) Different sources of ROS, including NOX, xanthine oxidase, iron catalysis, leukocytes, and mitochondria, contribute to myocardial stunning by inducing modifications to myofibrillar proteins (MPs) and altering the structure and function of sarcoplasmic reticulum (SR). (**B**) ROS can contribute to the development of myocardial stunning by its direct effect on the SR, depressing its Ca^2+^ transport and thereby leading to excitation–contraction uncoupling and cytosolic calcium overload. This can also lead to oxidative remodeling and proteolysis of MPs mediated through the activation of the calpain enzyme. (**C**) Myocardial stunning can also be a reason for depletion of myocardial high-energy phosphates produced by mitochondria, however, inotropic drugs like catecholamine or dopamine can enhance ATP generation and contractile function. Abbreviations: ROS, reactive oxygen species; SR, sarcoplasmic reticulum; NOX, nicotinamide adenine dinucleotide phosphate (NADPH) oxidase; ATP, adenosine triphosphate.

ROS is a collective term used for superoxide anion (O_2_^∙−^), hydroxyl radical (∙OH), hydrogen peroxide (H_2_O_2_), singlet oxygen (O^−^), and hypochlorous acid (HClO). RNS includes nitroxyl anion (NO^−^), nitrosonium cation (NO^+^), higher oxides of nitrogen, peroxynitrite (NO_3_^−^), S-nitrosothiols, and dinitrosyl iron complexes. When O_2_^∙−^ reacts with NO∙, it produces NO_3_^−^, which facilitates the oxidizing and nitrating reactions of biomolecules [45]. The exact time of generation during ischemia and reperfusion, characteristics, the cellular location and sources, the specific mechanism and reactions, and protective or detrimental levels of concentrations of ROS in the setting of myocardial stunning are complex and not completely understood [46]. An increasing number of studies have provided evidence that myocardial stunning may be mediated in large part by superoxide radical, H_2_O_2_, and ∙OH [47]. There are different sources of ROS such as nicotinamide adenine dinucleotide phosphate (NADPH) oxidase, xanthine oxidase, iron-catalyzed reactions, leukocytes, and mitochondria that can potentially contribute to ROS (Figure 1A), but the exact source of ROS contributing to myocardial stunning remains unclear. The role of leukocytes in ROS generation and myocardial stunning [48] is controversial [49] because myocardial stunning was successfully induced in saline-perfused hearts when perfusate did not contain any blood cells [50–52]. Xanthine oxidase is a source of ROS generation and contributes to myocardial stunning in rats and dogs [53,54]. However, its contribution to myocardial stunning in swine, rabbits, and humans is improbable since the myocardial expression of xanthine oxidase in these species is insignificant [55,56]. Increased NADPH oxidase (NOX) and ATPase activities were observed in mitochondria from stunned areas, indicating a disturbance in mitochondrial respiratory function in the stunned myocardium [57].

Furthermore, transgenic mice with cardiac-specific overexpression of GTP-binding protein RAC showed an increased NOX activity coupled with augmented myocardial stunning when exposed to 20 minutes of global ischemia and 45 minutes of reperfusion [58]. Iron-catalyzed reactions also play a significant role in the ROS generation and cardiac stunning development after a brief episode of reversible regional ischemia in dogs [41]. ROS cause oxidative modifications of vital biomolecules such as proteins, lipids, DNA, and RNA, consequently affecting cellular function. In myocardial stunning, the contractile apparatus and SR (Figure 1A) are very important, and ROS alter the structure and function of SR [59]. In addition, ROS induce oxidative modifications of myofibrillar proteins (MPs), specifically actin and tropomyosin (Figure 1A), in isolated saline-perfused rat heart after 30 minutes of global ischemia and 3 minutes of reperfusion. Indeed, actin and tropomyosin are essential proteins of contractile machinery [60]. ROS-induced oxidative modifications and myocardial stunning are largely attenuated by antioxidants such as SOD, catalase, the iron chelator desferrioxamine, or the xanthine oxidase inhibitor allopurinol and mercaptopropionylglycine or dimethylurea in experimental settings [4].

In recent years, our understanding of the role of ROS in myocardial ischemia and reperfusion has been further refined [61]. Heusch and colleagues [61] recently reviewed this topic in detail, emphasizing that ROS generation occurs predominantly during the early phase of reperfusion and arises mainly from mitochondria, NOXs, and xanthine oxidase. While excessive ROS formation contributes to oxidative injury and post-ischemic contractile dysfunction, a moderate and transient increase in ROS is essential for activating endogenous protective mechanisms such as ischemic preconditioning (IPC) and postconditioning. These insights highlight that ROS are not only mediators of injury but also important modulators of myocardial adaptation and survival, suggesting that future therapeutic approaches should aim to modulate rather than completely suppress ROS formation [61].

### Excitation–contraction coupling alterations in myocardial stunning

Fluctuation of intracellular free calcium concentration ([Ca^2+^]_i_), fluctuation of the contractile protein response to [Ca^2+^]_i_, and overloading are critical hallmarks that can affect myocardial contractile machinery [35]. In post-ischemic reperfusion, myocytes are not only subjected to ROS burst but also cytosolic Ca^2+^ overload induced by increased sodium–proton exchange and a consequent reverse-mode sodium–calcium exchange [11,13]. The ROS depress SR Ca^2+^ transport and also contribute to cytosolic Ca^2+^ overload [59] (Figure 1B). Whether cytosolic Ca^2+^ overload is the primary culprit of myocardial stunning is not appreciated since calcium antagonists, which further increase cytosolic Ca^2^ levels, were administered either before ischemia or just before reperfusion and attenuated myocardial stunning in *ex vivo* and *in vivo* settings [12]. Over the years, research has been overwhelmingly focused on myofilaments as a binding site involved in myocardial stunning in *ex vivo* heart models. Kusuoka et al. [31], for the first time, found impaired myofilament function (depressed maximal calcium-activated pressure) in stunned ferret hearts. Marbán et al. [62,63] demonstrated by NMR spectroscopy that myocardial stunning results from reduced calcium response.

Further, calcium sensitivity and maximal calcium-activated force contribute to calcium responsiveness, representing a fractional response to induce calcium levels high to that maximum force [35]. Other studies on ventricular trabeculae from rat hearts and in isolated perfused ferret hearts confirmed that calcium transient was not reduced; only both maximum contractile force and calcium sensitivity were reduced [31,64]. Experiments by Heusch et al. [1] in a porcine model of myocardial stunning concluded that the stunned myocardium remained responsive to changes in extracellular calcium, suggesting that calcium availability is not limited in stunned myocardium, but maximal calcium responsiveness was reduced. It implies that the underlying mechanism of excitation–contraction uncoupling prevails distal to calcium availability at the level of the contractile proteins [1]. Therefore, reduced calcium responsiveness has been attributed to oxidative remodeling and proteolysis of MPs through activation of enzymes like calpain by calcium overload [4,35,60]. Gao et al. [65] found thin filament protein troponin I (TnI) degradation in an *ex vivo* myocardial stunning rat model, certainly providing an attractive illustration of myocardial stunning. Accordingly, selective overexpression of proteolytic TnI induces a myocardial stunning-like phenotype with ventricular dilation, impaired contractility, and decreased calcium responsiveness in mice [66]. However, a study by Feng et al. [67] demonstrated no causal link between TnI proteolysis and the myocardial stunning after a brief ischemic episode. TnI degradation is caused by other mechanisms such as preload-induced proteolysis of TnI rather than ischemia [67,68]. ROS generation could be viewed as the first main event contributing to cytosolic calcium overload by provoking injury to the SR and impaired calcium responsiveness by oxidative modification of MPs [59,60] (Figure 1B). Consequently, cytosolic calcium overload causes mitochondrial dysfunction, leading to further ROS generation. Recent data on quantitative proteomic and phosphor-proteomic profiling of ischemic myocardial stunning in swine identified principal protein networks implicated in myocardial stunning, including contractile protein dysfunction, ECM protein degradation, and induction of apoptosis [15].

## The histological perspective of myocardial stunning

Histological analysis of stunned myocardium by triphenyl tetrazolium chloride stunning or hematoxylin/eosin staining in experimental animal models has shown no anatomic evidence of necrosis [8,69–71]. Electron micrographs prepared from stunned myocardial biopsies revealed abundant glycogen, homogeneously distributed chromatin, and rare formation of intracellular vacuoles [69]. The most striking finding observed in this study was the significant unfolding of mitochondrial cristae, described as an energized, twisted, or condensed configuration [69]. Further studies on experimental dog models of myocardial stunning reported minor structural abnormalities in stunned myocardium, such as rare cytoplasmic vacuoles, moderate depletion in glycogen, mild intermyofibrillar edema, myofibrillar relaxation with broad I bands, and nuclear chromatin clumping [8,71]. No ultrastructural changes of irreversible nature have been observed, including amorphous mitochondrial dense bodies, sarcolemmal breaks, marked intracellular edema, or mitochondrial damage [71]. Serum cardiac troponins (cTn) elevations have been considered indicative of myocardial injury leading to cardiomyocyte necrosis and are currently the most preferred diagnostic marker for detecting MI in the clinic [72]. A recent study in a porcine model of myocardial stunning demonstrated a delayed release of cTnI, which exceeded the 99th percentile after 60 minutes of reperfusion and was readily detectable after 24 hours. Although histological analysis at 60 minutes did not provide any evidence of necrosis, the TUNEL (Terminal deoxynucleotidyl transferase dUTP Nick-End Labeling) assay identified myocytes undergoing apoptosis, which was absent after 24 hours of reperfusion, suggesting that cTnI increase occurs after ischemia of brief duration that is insufficient to produce myocardial necrosis and represent myocardial injury related to apoptosis in the absence of pathological evidence of necrosis [73]. Supporting these findings, our study found that both hs-cTnI and hs-cTnT levels increase similarly after short periods of ischemia that do not result in overt necrosis. However, the hs-cTnI/hs-cTnT ratio tends to rise following longer periods of ischemia that cause significant necrosis. A low hs-cTnI/hs-cTnT ratio, around 1, may indicate cTn release without necrosis [74].

Ultrastructural studies on the extracellular collagen matrix of stunned myocardium showed that the myocardial collagen matrix is critically damaged in stunned myocardium, which might explain the greater myocardial compliance and ineffective contractibility of the stunned myocardium [71,75].

## Metabolic alterations in myocardial stunning

It is now appreciated that brief myocardial ischemia not only induces reversible contractile dysfunction of the myocardium but also produces an array of metabolic derangements such as a reduction in energy-rich phosphate compounds and a decrease in adenine nucleotides and glycogen, as well as the accumulation of breakdown products of energy-providing substrates [50,76–78]. It was believed that the myocardium stunning is a consequence of the insufficient generation of adenosine triphosphate (ATP) (Figure 1C) by the myocardium to support the expected workload [7]. The observation that brief ischemic exposures lead to depletion of myocardial high-energy phosphates (Figure 1C), and these compounds were slowly repleted in a similar time course as recovery in myocardial function, might provide support for this theory [8,79–81].

Furthermore, it was proposed that increasing the myocardial energy demand of the post-ischemic heart with inotropic drugs such as catecholamine (Figure 1C) could be a better way to assess the relevance of myocardial energy metabolism in stunned myocardium [76]. Suppose ATP synthesis was impaired to limit baseline myocardial function [76], inotropic stimulation should either not improve the function or improve the function followed by deterioration due to the utilization of high-energy phosphate metabolites with a greater rate than resynthesizes [76,82]. On the other hand, if the mitochondrial function were intact, ATP generation should be enhanced with inotropic stimulations with preserved high-energy phosphate. Several studies have shown that inotropic stimulation can enhance contractile function in stunned myocardium (Figure 1C), suggesting metabolic reserves [83–85]. However, in these studies, high-energy phosphates were not measured directly. Arnold et al. [86] investigated the relationship between dopamine infusion and high-energy phosphates in an experimental dog model of regional ischemia following 2 hours of reperfusion. Although dopamine infusion reversed the regional ischemic dysfunction (Figure 1C), biopsies collected from mid and endomyocardial showed a 32% reduction in phosphocreatine and 37% reduction in ATP. However, such results were challenging to explicate since collected samples were mixed of injured and stunned myocardium. The moderate reduction in high-energy phosphate could either be derailed metabolic changes in stunned myocytes or a significant decrease in ATP and phosphocreatine in severely injured myocytes masking the overall picture of well-preserved stunned myocytes in the same sample.

To overcome such technical challenges, Ambrosio et al. [76] studied myocardial high-energy metabolism in post-ischemic stunned hearts, and the high energy demand was induced by isoproterenol administration in an *ex vivo* rat model. They concluded that global ischemia could induce myocardial stunning and myocardial ATP depletion in the absence of histological damage in isolated, not-blood-perfused rat hearts exposed to global ischemia. However, the myocardial ATP levels and phosphocreatine were not decreased during increased energy demand [4].

Despite these abnormalities, the stunned myocardium sustains a significant increase in function and energy demand upon inotropic stimulations, indicating that neither the contractile engine nor the mechanism for the generation of ATP was severely damaged in the stunned myocardium [4,76]. Moreover, several findings support the notion that prolonged nucleotide depletion is not the underlying mechanism of myocardial stunning since creatine phosphate levels were sharply restored, and no correlation between ATP depletion and functional impairment was observed after regional reversible ischemia, suggesting that stunned myocardium preserves oxidative metabolism and mitochondrial function [50,77,81]. Indeed, adenosine infusion accelerated ATP repletion, but this elevation of ATP levels did not improve reduced regional contractility during reperfusion [87,88].

## Potential epigenetic regulation of myocardial stunning

**Figure 2:**
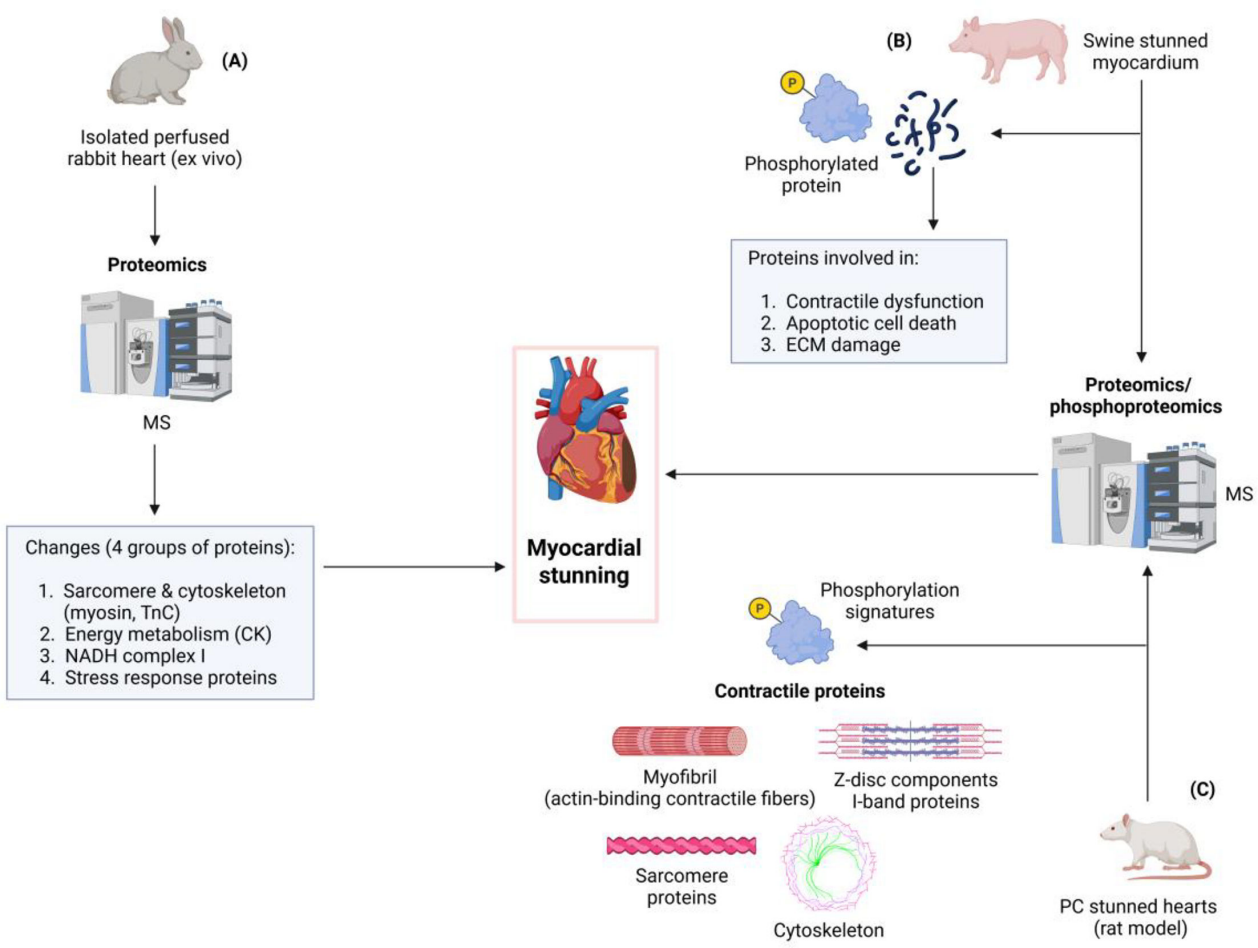
Established work and mechanisms in myocardial stunning. (**A**) A proteomics study on an isolated perfused rabbit heart showed changes in sarcomere and cytoskeleton proteins, NADH complex I, energy metabolism, and stress response proteins contributing to myocardial stunning. (**B**) Changes in phosphorylation signatures of proteins involved in contractile dysfunction, ECM damage, and myocyte apoptosis were identified in stunned myocardium in swine. (**C**) Recent results from our lab indicated that ischemic preconditioning exacerbates post-ischemic myocardial stunning, accompanied by changes in phosphosites of proteins involved in contractile function, including sarcomere proteins, Z-disc, I-band, contractile fibers, myofibril, and cytoskeleton. Abbreviations: MS, mass spectrometry; ECM, extracellular matrix; CK, creatine kinase; NADH complex I, nicotinamide adenine dinucleotide (NAD) + hydrogen (**H**) ubiquinone oxidoreductase complex I; PC, preconditioned.

Epigenetics is a rapidly growing research field that investigates changes in gene expression due to chemical alterations in DNA and its associated proteins without altering the DNA sequence [18,19]. Gene regulation by epigenetic mechanisms is classified into three categories: DNA methylation, histone modification, and ncRNA [89] (Figure 2A). DNA methylation occurs at the fifth carbon atom of cytosine (5mC), thereby regulating gene transcription. This regulation primarily depends on CpG islands, which are abundant in 60% of the promoter regions in the human genome [90]. DNA hypermethylation by DNA methyltransferases (DNMTs) maintains the heterochromatin structure and inhibits gene expression [91]. Histone modification includes methylation, acetylation, ubiquitination, and phosphorylation on histone tails [18]. Among these, acetylation and methylation have been studied extensively. These modifications reprogram the chromatin architecture and gene expression patterns [92]. Histone methylation by histone methyltransferases can lead to the formation of euchromatin or heterochromatin, thereby activating or repressing gene transcription depending on the amino acid residues being methylated [93]. Conversely, histone demethylation by histone demethyltransferases leads to the formation of an open chromatin structure and activation of gene transcription [94]. Histone acetylation by histone acetyltransferases and deacetylation by histone deacetylases often results in open or compact chromatin structure, causing gene transcription or silencing, respectively [95,96]. ncRNAs are RNA molecules that do not encode proteins but play a regulatory role in modulating gene expression [97]. MicroRNAs (miRNAs) and long noncoding RNAs are two subclasses of ncRNAs implicated in the development and progression of several cardiovascular diseases (CVDs) [98,99].

Enzyme-mediated epigenetic modifications are reversible (Figure 1A) and have been shown to influence the expression of genes involved in CVDs [100] (Figure 2A). Understanding these changes in gene regulation is crucial for uncovering mechanisms underlying cardiac pathologies and dysfunction and providing an opportunity for reprogramming [101]. A myocardium is defined as ‘stunned’ when it suffers from reversible contractile dysfunction induced by acute ischemia, which persists even after the complete restoration of blood supply without any irreversible histological damage [102]. Given that both epigenetic mechanisms and myocardial stunning are reversible processes (Figure 1A), there is a high possibility that epigenetic mechanisms significantly contribute to the development of myocardial stunning. Notably, the mechanisms associated with myocardial stunning, as highlighted in this review, are influenced by epigenetic modifications occurring during ischemia-reperfusion [16,17,103,104]. Indeed, myocardial stunning is considered a form of ischemia-reperfusion injury [105]. For instance, ROS can either trigger or be influenced by epigenetic modifications, resulting in cardiac dysfunction during ischemia-reperfusion injury [106]. Studies have shown that targeting global DNA methylation in I/R hearts using its inhibitor significantly reduces the I/R-associated infarct size by 45% and improves dysferlin levels coded by DYSF gene. This modulation affects genes involved in cell death apoptotic pathways (*CASP3*, *CASP7*, and *PARP*), inflammation (*IL-1β, TLR4, ICAM1, and MyD88*), oxidative stress (*SOD1*, Catalase, *GPX2*, and *NFkB*), and mitochondrial function and regulation (MT-*ND1, ND3, COX1, ATP6, PGC1α*, and *TFAM*) in cardiac tissue [107].

**Figure 3:**
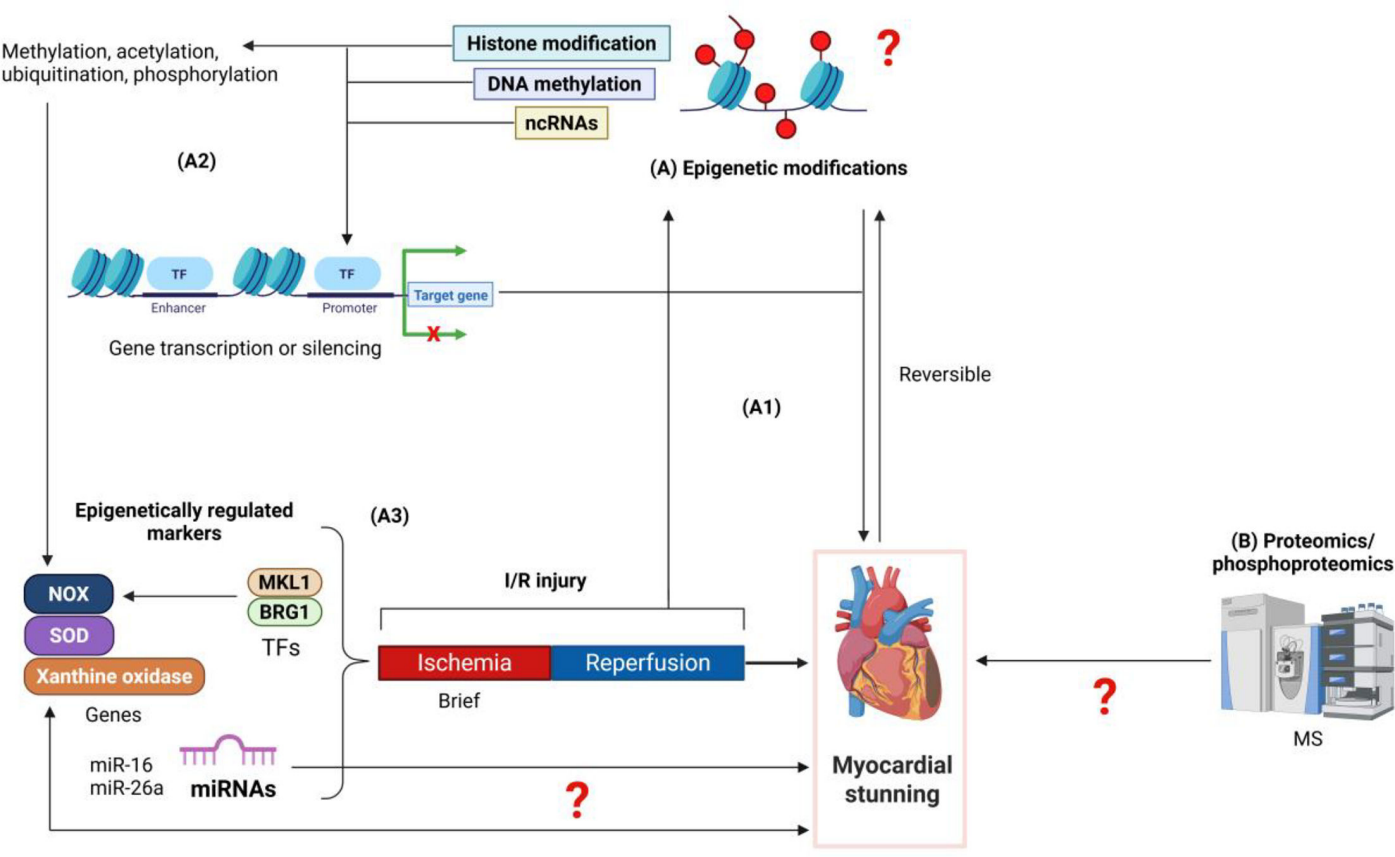
Speculative mechanisms in myocardial stunning. (**A**) Epigenetic modifications, including histone modification, DNA methylation, and non-coding RNAs, are reversible and can influence gene expression in cardiovascular disease (**A1, A2**). These mechanisms remain unknown in myocardial stunning, a reversible cardiac dysfunction. Histone modifications such as methylation, acetylation, ubiquitination, and phosphorylation can drive the gene expression of NOX, SOD, and xanthine oxidase, which are regulated during ischemia-reperfusion (I/R) injury by transcription factors like MKL1 and BRG1 (**A3**). However, these have not been studied in myocardial stunning. The role of miRNAs, such as miR-16 and miR-26a, has only been explored in takotsubo syndrome, hypothesized as a form of myocardial stunning (**A3**). The involvement of these non-coding RNAs in myocardial stunning remains unknown. (**B**) Proteomics and phosphor-proteomics following I/R injury are powerful tools for understanding the pathophysiology of myocardial stunning but remain incompletely understood and require further investigation. Abbreviations: I/R injury, ischemia-reperfusion injury; TFs, transcription factors; MKL1, megakaryoblastic leukemia 1; BRG1, chromatin remodeling protein BRG1; NOX, nicotinamide adenine dinucleotide phosphate (NADPH) oxidase; SOD, superoxide dismutase; miRNAs, microRNAs; MS, mass spectrometry.

Other studies on myocardial I/R injury revealed that the regulation of NOX [106] and xanthine oxidase gene expression is driven by epigenetic regulatory mechanisms [108] (Figure 3A). However, this has not been studied in the context of myocardial stunning. For example, Liming Yu et al. showed that myocardin-related transcription factor A, also known as megakaryoblastic leukemia 1, is increased in I/R injury and involved in the transcriptional activation of NOX genes (*NOX1*, *NOX2*, and *NOX4*) in macrophages by binding to the promoters of these NOX genes and recruiting H4K16 acetyltransferase and other active histone acetylation marks, accompanied by open chromatin structures of the promoters [103]. Similarly, Zilong Li et al. reported the role of the chromatin remodeling protein BRG1 in cardiac I/R injury [104]. The study showed that in the setting of hypoxia-reoxygenation, BRG1 may link epigenetic activation of NOX transcription in endothelial cells to cardiac I/R injury. Suppression of NOX trans-activation by BRG1 silencing was accompanied by the loss of active histone modifications (H3 and H4 acetylation) and the recruitment of repressive histone modification (H3K9 histone demethylation) [104]. While no studies have yet addressed the role of epigenetics in myocardial stunning, research has been limited to investigating the role of ncRNAs in takotsubo syndrome (TTS). Many researchers hypothesize that TTS is a form of myocardial stunning characterized by reversible ventricular dysfunction and clinical features similar to MI [109,110]. For example, Liam S. Couch et al. explored the role of miR-16 and miR-26a (Figure 3A) as stress and anxiety markers that can sensitize the heart to TTS-like changes induced by adrenaline. These microRNAs could provide a mechanism for an increased likelihood of TTS [17]. Another study showed that the regulated expression of four stress- and depression-related circulating miRNAs (miR-16, miR-26a, miR-1, and miR-133a) could serve as robust biomarkers to distinguish takotsubo cardiomyopathy from acute MI [ST-elevation MI (STEMI)] patients [16]. Further investigations are needed to clarify the role of miRNAs as prognostic and diagnostic biomarkers of myocardial stunning and to differentiate myocardial stunning from TTS.

The epigenetic regulatory mechanisms addressed in I/R injury, such as those involving NADPH and xanthine oxidase, represent significant markers due to their role in ROS generation and cardiac muscle dysfunction. Investigating these mechanisms in the context of myocardial stunning is of great importance, as it could provide new insights into therapeutic strategies for this condition.

## Proteomics and phosphor-proteomics in myocardial stunning

The importance of proteomics and phosphor-proteomics, especially as unbiased approaches, is paramount in understanding the pathophysiology of myocardial stunning. These advanced techniques enabled a comprehensive and unbiased study of changes in proteins and phosphoproteins following I/R injury [111]. A notable study utilized ion current-based proteomics and quantitative phosphor-proteomics to explore alterations in protein expression and phosphorylation in swine hearts, uncovering changes linked to contractile dysfunction, ECM damage, myocyte apoptotic cell death, and protein–protein interactions [15] (Figure 1B). Altered phosphosites were mediated by protein kinase C phosphorylation of cTn I-S199 and CaMKII-mediated phosphorylation of phospholamban-T17 [15]. This study advances the understanding of molecular signatures associated with ischemic myocardial stunning and offers a well-annotated database of the swine heart proteome within a clinically relevant animal model. Nevertheless, future research should prioritize elucidating the mechanistic aspects of these changes to determine if they can alter the stunning phenotype and potentially be translated into clinical practice.

Taking into account the hypothesis that delayed recovery of contractile function in stunned myocardium is attributed to impaired Ca^2+^ sensitivity of myofilaments and damage in one or few sarcomeric proteins as a result of ischemia-reperfusion injury [31,64], this does not explain the physiological changes observed after myocardial stunning, including myocardial oxygen consumption and ATP demand.

A proteomics study by White M.Y. et al. on an isolated perfused rabbit heart following 15 minutes or 60 minutes of ischemia showed changes in proteins from four functional groups, including (i) sarcomere and cytoskeleton, in particular myosin light chain-2 and troponin C; (ii) redox regulation of NADH ubiquinone oxidoreductase complex; (iii) energy metabolism, including creatine kinase; and (iv) stress response [112] (Figure 2B). Changes in these proteins appeared to be the result of isoelectric point shifts resulting from chemical modifications and molecular mass shifts resulting from proteolytic or physical fragmentation, supporting the notion that the time course for the onset of injury associated with myocardial stunning is too brief to induce changes in a single protein but rather a functional impairment in the proteome [112]. However, this study used an isolated perfused rabbit heart model, which may not fully replicate the *in vivo* physiology of myocardial stunning. Additionally, the methods employed may miss certain protein modifications and low-abundance proteins. Comparative gel analysis introduces subjectivity, and functional implications of protein changes remain unexplored. Further, *in vivo* studies are needed to confirm findings and understand clinical relevance.

Building on this foundation, in our recent study, we utilized phosphor-proteomic analysis to examine the effects of IPC on myocardial contractile function in rat hearts (Figure 3B). We found that IPC enhances post-ischemic myocardial stunning and induces notable alterations in phosphorylation sites of crucial proteins involved in myocardial contraction, including those associated with the sarcomere, Z-disc, I-band, contractile fibers, myofibrils, and the cytoskeleton (Figure 3B). Although a direct causal link between the intensified stunning and reduced infarct size post-preconditioning has not been established, our analysis suggests that IPC may confer cardioprotective effects by modulating contractile activities. Specifically, our phosphor-proteomic analysis identified changes in sarcomere proteins, Z-disc components, I-band proteins, and actin-binding contractile fibers (Figure 3B), primarily localized within critical subcellular structures essential for cardiac function [14]. Further investigation is required to fully understand the functional relevance of these phosphorylation signatures.

## Myocardial stunning within the framework of ischemic preconditioning and cardioprotection

Brief, transient episodes of myocardial ischemia preceding a prolonged ischemic insult, referred to as myocardial IPC, reduce infarct size in experimental models [113]. Nevertheless, the mechanisms underlying this protection remain incompletely understood and have yet to be consistently reproduced in clinical studies. Although both IPC and myocardial stunning arise from short periods of ischemia, they have traditionally been regarded as separate phenomena [7,114].

Myocardial stunning has been viewed as a manifestation of ischemia-reperfusion injury [4,35], but its physiological significance remains uncertain. IPC was initially suggested to attenuate or prevent stunning; however, several studies have failed to show a consistent reduction in post-ischemic dysfunction following IPC [115]. Other investigations demonstrated that stunning develops early during ischemia, before major depletion of myocardial energy stores [116]. As most myocardial energy is utilized for contraction, the reversible depression of contractility may preserve energy for essential cellular processes and protect against irreversible injury [116,117].

Heusch [118] discussed stunning within the broader context of ischemia-reperfusion and cardioprotection. He proposed that conditioning does not abolish stunning but shifts the outcome of ischemia from irreversible necrosis to reversible dysfunction. This shift reflects activation of intrinsic protective pathways that reduce calcium overload, oxidative stress, and microvascular obstruction during reperfusion. The persistence of stunning after preconditioning therefore indicates conversion of irreversible injury into a reversible state and represents a marker of myocardial salvage [118].

In line with this concept, our study showed that IPC accentuated reversible post-ischemic akinesia while reducing infarct size [14]. Reversible dysfunction after prolonged ischemia was observed predominantly in preconditioned rats, indicating that IPC promotes reversible rather than irreversible impairment. Phospho-proteomic analysis revealed altered phosphorylation of proteins related to sarcomeric structure, Z-disc, and I-band, including those involved in actin binding and cytoskeletal organization [14]. These changes suggest that controlled modulation of contractile protein phosphorylation may transiently suppress contraction to conserve energy and facilitate recovery during reperfusion [14].

In a subsequent proteomic study [119], IPC was associated with distinct protein expression profiles compared with non-preconditioned ischemia-reperfusion. IPC activated endocytic and Fc gamma receptor-mediated phagocytosis pathways at early time points and down-regulated proteins related to tissue remodeling, inflammation, and coagulation at later stages [119]. In contrast, non-preconditioned myocardium exhibited increased expression of inflammatory and injury-related proteins [119]. These findings indicate that IPC initiates early protective and immune-modulatory responses that limit irreversible injury and support reversible contractile dysfunction consistent with myocardial stunning [119].

Furthermore, our recent transcriptomic and epigenetic study [120] showed that IPC reprograms the cardiac transcriptome through a dynamic DNA methylation study [120]. IPC reduced global DNMT activity and induced hypomethylation of cardioprotective genes such as *Cebpd*, *Nfkbia*, *Gadd45b*, *Jun*, and *Aplod1*, while hypermethylating maladaptive genes including *Tmem200c* and *Fgfr4* study [120]. These molecular adaptations were associated with reversible post-ischemic dysfunction and reduced necrosis, supporting that IPC engages multiple regulatory levels that favor reversible over irreversible injury study [120].

Collectively, these findings indicate that myocardial stunning is not solely a manifestation of injury but represents a reversible and adaptive component of cardioprotection. Consistent with Heusch’s concept, IPC does not prevent stunning but modifies its functional and molecular characteristics. The combined functional, proteomic, and transcriptomic data suggest that IPC induces co-ordinated post-translational and epigenetic remodeling that may transiently suppress contractility, conserve energy, and promote myocardial recovery during reperfusion.

## Clinical relevance of myocardial stunning

Although myocardial stunning was regarded as an experimental entity when discovered, over the years, it has been appreciated that the phenomenon is relevant clinically [121]. With the advancements in reperfusion therapies in the clinic, it has become clear that myocardial stunning is intrinsic to CAD and may contribute to associated complications [121]. Other clinical events such as unstable angina, coronary spasm, coronary artery bypass surgery, and exercise-induced ischemia can also result in myocardial stunning in patients with stable angina and CAD [4,122]. While diagnosing myocardial stunning, it is imperative to demonstrate that the contractile dysfunction is entirely reversible and occurs under normal coronary blood flow, as demonstrated by Bolli et al. [123]. Elective PCI is a standard setting used to observe transient contractile dysfunction and regional myocardial blood flow over time in the patients [4]. Wdowiak‐Okrojek et al. [124] recruited 97 MI patients who successfully underwent PCI and followed their cardiac function with serial 2‐dimensional echocardiographic speckle tracking over 180 days of reperfusion. They reported that the most significant recovery in regional systolic function occurs in the first two days of reperfusion. There has been further improvement in systolic function on day three to day 180, but not as profound as in day one to day two. However, the diastolic improvement in function took time, and the marked recovery in diastolic function was observed in the first seven days. Indeed, this study reported both systolic and diastolic stunning in recent years. In another study, gated single‐photon emission computed tomography myocardial perfusion imaging was performed on 120 acute MI patients who underwent PCI before and after 6 months of hospital discharge. They showed that the Left ventrical ejection fraction (LVEF) before discharge was 47% and improved to 51% 6 months after the discharge with an increase in EF > 5 units in 54 patients. A significant correlation between LVEF recovery and the amount of salvaged myocardium was also observed [125]. Several other studies reported the existence of myocardial stunning in patients with STEMI following PCI [126]. Angioplasty balloon inflation for a brief duration in the coronary artery has been shown to induce myocardial stunning in patients undergoing elective PCI [127]. McCormick et al. [127] studied a cohort of 20 patients undergoing elective PCI with single-vessel CAD and preserved LV function. The pressure‐volume loops and hemodynamics were measured to assess the LV function by inserting a conductance catheter into the LV cavity at baseline, during the balloon inflation, and after 30 minutes of balloon deflation. They observed a reduced LV function in terms of reduced cardiac output, EF, dP/dT max, and elevated Tau, which were consistent with myocardial stunning. Of interest, patients pre-treated with glucagon‐like peptide‐1 showed protection against ischemia-induced LV dysfunction and myocardial stunning. Jeroudi et al. [128] observed myocardial stunning in patients with unstable angina; however, this evidence was suggestive since they did not document the normal blood flow. Myocardial stunning has been shown to occur after global ischemic cardioplegic arrest, but it is difficult to distinguish true myocardial stunning from contractile dysfunction induced by other factors such as temperature alterations, ionic concentrations, and loading conditions in this setting [4,122,123]. Since its first description in experimental models, the clinical importance of myocardial stunning has remained debated. Heusch [129] questioned whether myocardial stunning should be regarded as a distinct clinical entity, arguing that isolated reversible dysfunction with fully restored perfusion is rarely seen in patients [129]. He emphasized that most clinical scenarios interpreted as ‘stunning,’ such as transient dysfunction after PCI or brief ischemia, coexist with microvascular obstruction, edema, or incomplete reperfusion [129]. In contrast, Menasché and Galiñanes [130] highlighted that transient left ventricular dysfunction consistent with myocardial stunning is a frequent observation after cardiac surgery and cardioplegic arrest (PMID: 9689441). They pointed out that this reversible dysfunction often occurs despite adequate myocardial protection and normal coronary flow, suggesting additional mechanisms such as inflammatory activation, oxidative stress, and calcium-handling disturbances during reperfusion [130]. Clinically, this postoperative stunning may prolong recovery and require short-term inotropic or mechanical support [130]. These findings underline that myocardial stunning is not only experimentally relevant but also of direct practical importance in surgical settings [130].

The National Heart, Lung, and Blood Institute (NHLBI) Workshop on *Stunning, Hibernation, and Preconditioning* [131] placed these observations into a broader translational framework, concluding that myocardial stunning represents a fundamental manifestation of reperfusion injury occurring across a continuum of clinical ischemic conditions [131]. The workshop emphasized its significance for interpreting delayed functional recovery after reperfusion, optimizing cardioprotective interventions, and distinguishing viable but dysfunctional myocardium from infarcted tissue [131]. Supporting this view, Ehring et al. showed that the Angiotensin-Converting Enzyme (ACE) inhibitor ramiprilat attenuates myocardial stunning through a bradykinin-prostaglandin-dependent signaling pathway, demonstrating that the process can be pharmacologically modified and therefore clinically relevant [132].

Modern clinical imaging and physiological studies further substantiate the occurrence of myocardial stunning in patients undergoing reperfusion therapy [133]. Serial echocardiographic and magnetic resonance assessments after PCI have demonstrated progressive recovery of regional wall motion and global systolic function, proportional to the extent of salvaged myocardium [134]. The ST-Elevation Myocardial Infarction (STAMI) study confirmed this pattern in patients with anterior STEMI, showing gradual recovery of systolic function over days to weeks, with faster improvement in women than in men, suggesting sex-related differences in post-ischemic recovery [135]. These findings indicate that reversible post-ischemic dysfunction remains an integral part of the clinical spectrum of myocardial reperfusion.

Accurate diagnosis of myocardial stunning remains challenging because it requires demonstration of normal perfusion and reversible dysfunction, criteria that are typically confirmed only after recovery. As discussed by Sharif [134], advanced imaging techniques such as cardiac magnetic resonance, positron emission tomography, and single-photon emission computed tomography allow differentiation between stunned, hibernating, and necrotic myocardium by identifying normal perfusion within hypocontractile segments [134]. Low-dose dobutamine echocardiography and stress cardiac magnetic resonance can further detect preserved inotropic reserve in viable myocardium [134]. More recently, Doppler-based indices such as the ‘myocardial stunning index,’ derived from coronary flow velocity immediately after reperfusion, have been proposed as potential prospective markers of reversible dysfunction [134]. These diagnostic developments are clinically relevant, as early identification of stunned but viable myocardium facilitates appropriate hemodynamic support and targeted cardioprotective therapy during the reperfusion phase [134].

Overall, the controversy surrounding the clinical importance of myocardial stunning largely reflects differences in its interpretation rather than its existence. While rarely isolated as a pure entity, myocardial stunning represents a reproducible physiological response of viable myocardium to ischemia and reperfusion. Its recognition in clinical settings helps explain why the recovery of contractile function lags behind restoration of blood flow and underscores the importance of managing reperfusion injury to optimize functional recovery. Furthermore, the great importance of myocardial stunning lies in the gradual recovery of myocardial function after reperfusion in the setting of non-transmural MI [125,126,136]. However, distinguishing true myocardial stunning in its narrow perspective from the resolution of edema and inflammation and from infarct healing, myocardial remodeling, and angiogenesis is impracticable [4].

## Therapeutic approaches to myocardial stunning

When myocardial stunning is recognized as the cause of mild post-ischemic contractile dysfunction, it is usually transient and well tolerated, and no specific therapy is required [137]. However, in cases of severely depressed cardiac function or in high-risk clinical settings, post-ischemic dysfunction may contribute to hemodynamic instability and adverse outcomes, requiring active management. In such situations, the therapeutic goal is to support myocardial performance while limiting further adverse consequences of myocardial stunning, such as heart failure [137].

Inotropic agents such as dopamine, epinephrine, and isoproterenol can transiently restore contractile function by increasing intracellular calcium availability and enhancing myofilament sensitivity [121,137,138]. However, these agents also increase myocardial oxygen consumption and may exacerbate oxidative stress, arrhythmias, or microvascular injury, particularly when administered repeatedly or at high doses [138]. Data from the SWEDEHEART registry, a nationwide cohort including over 16,000 patients with cardiogenic shock, showed that inotrope use was independently associated with higher 30-day mortality, even after comprehensive adjustment for confounders [139]. This finding underscores the narrow therapeutic window of conventional inotropes: while they may transiently improve hemodynamics in stunned myocardium, prolonged stimulation of contractility in metabolically compromised tissue may worsen reperfusion injury and delay recovery.

Among other pharmacological options, levosimendan, a calcium-sensitizing inotrope, provides inotropic and vasodilatory effects without increasing myocardial oxygen demand or inducing arrhythmias [138]. By stabilizing the interaction between calcium and troponin C and opening ATP-sensitive potassium channels, levosimendan improves contractility and reduces afterload, thereby enhancing cardiac output while limiting metabolic stress on stunned myocardium [138]. Its combined hemodynamic and cardioprotective profile makes it particularly useful in settings of reversible post-ischemic dysfunction and perioperative myocardial stunning [138,140].

In addition to inotropic agents, several drug classes act directly on the pathophysiological mechanisms of stunning. Calcium-channel antagonists, when administered before or during early reperfusion, attenuate intracellular calcium overload and accelerate recovery of contractile function [141,142]. ACE inhibitors improve post-ischemic recovery through bradykinin-prostaglandin-dependent pathways that enhance endothelial and microvascular function, whereas adenosine reduces reperfusion-induced oxidative stress and inflammation [143,144]. These interventions act at the level of cellular signaling and microcirculatory stability, promoting the recovery of viable myocardium without imposing excessive energetic demand [143,144].

Experimental evidence supports that myocardial stunning can be attenuated not only by transient inotropic stimulation but also by pharmacological interventions targeting calcium handling and endothelial signaling. Calcium-channel antagonists such as nisoldipine and diltiazem improved post-ischemic recovery by limiting calcium overload and hypercontracture [142,145]. Similarly, the ACE inhibitor ramiprilat enhanced recovery in reperfused dog hearts through a bradykinin-prostaglandin-dependent mechanism, improving microvascular perfusion and reducing oxidative stress [132]. These findings indicate that modulation of calcium influx and endothelial function can directly influence the severity and duration of myocardial stunning. Together, these pharmacological insights reinforce that myocardial stunning represents a dynamic and modifiable process rather than an irreversible form of myocardial injury.

For most patients, pharmacological response and spontaneous recovery of the stunned myocardium are favorable, and function normalizes within hours to days. Nonetheless, identifying cases where stunning contributes to clinically significant dysfunction, particularly in patients with compromised reserve, is essential. Careful use of hemodynamic support, avoidance of excessive inotropic stimulation, and timely administration of agents that stabilize reperfusion biology remain the most effective therapeutic principles.

## Conclusion and Future Directions

The exploration of myocardial stunning has significantly advanced our understanding of cardiac pathophysiology, yet several avenues remain open for future research. Addressing these gaps will enhance our ability to mitigate myocardial stunning’s clinical impact and improve patient outcomes.

### Advanced proteomic and phospho-proteomic analysis

Future studies should leverage advanced proteomic and phospho-proteomic technologies to map the dynamic changes in protein expression and phosphorylation with higher resolution and precision. High-throughput mass spectrometry and bioinformatics tools can identify novel biomarkers and therapeutic targets associated with myocardial stunning. Investigating the temporal sequence of these molecular events can also provide insights into the progression and resolution of stunning, aiding in the development of timely therapeutic interventions.

### Epigenetic mechanisms and therapeutic potential

The role of epigenetic modifications in myocardial stunning is a promising but underexplored area. Future research should focus on delineating the specific epigenetic changes that occur during myocardial stunning and their functional consequences on myocardial cells. Studies employing DNA methylation inhibitors, histone modification modulators, and ncRNA-based therapies could reveal new strategies for preventing or reversing myocardial stunning. Moreover, understanding the interplay between epigenetic modifications and other cellular pathways involved in myocardial stunning could uncover synergistic therapeutic approaches.

### Clinical translation of experimental findings

Bridging the gap between experimental models and clinical practice is crucial for translating research findings into effective treatments. Large-scale clinical trials should be designed to evaluate the efficacy and safety of potential therapeutic interventions identified through proteomic, phospho-proteomic, and epigenetic studies. These trials should include diverse patient populations to ensure generalizability and address potential variability in treatment responses.

### Non-invasive diagnostic tools

Developing non-invasive diagnostic tools for early detection and monitoring of myocardial stunning is another critical area for future research. Advances in imaging technologies, such as cardiac magnetic resonance imaging (MRI) and positron emission tomography (PET), combined with molecular biomarkers identified through proteomic and phospho-proteomic analyses, could provide real-time insights into myocardial function and the extent of stunning. Such tools would enable personalized treatment strategies and improve patient management.

### Understanding the role of reactive oxygen species (ROS)

While the involvement of ROS in myocardial stunning is well-documented, the precise sources and mechanisms of ROS generation remain incompletely understood. Future research should aim to identify the specific cellular sources of ROS and elucidate their interactions with other molecular pathways during ischemia-reperfusion injury. Targeting these sources with novel antioxidants or ROS-modulating therapies could offer new avenues for protecting the myocardium from stunning.

### Exploring the impact of ischemic preconditioning (IPC)

IPC has shown potential in modulating the effects of myocardial stunning. Further studies are needed to understand the molecular mechanisms by which IPC influences protein phosphorylation and gene expression. Investigating the long-term effects of IPC on myocardial function and its potential to enhance cardiac resilience against subsequent ischemic events could provide valuable insights for clinical applications.

### Integrative approaches and systems biology

Employing integrative approaches that combine proteomics, phospho-proteomics, epigenomics, and transcriptomics will provide a holistic view of the molecular alterations in myocardial stunning. Systems biology approaches can model the complex interactions between various molecular pathways and predict the effects of potential therapeutic interventions. Such comprehensive analyses will facilitate the identification of key regulatory nodes and the development of multifaceted treatment strategies.

By addressing these future directions, we can continue to unravel the complexities of myocardial stunning and translate these findings into clinical practice, ultimately improving outcomes for patients suffering from ischemic heart disease.

## Abbreviations

ATP: adenosine triphosphate
CABG: coronary artery bypass graft
CAD: coronary artery disease
CVDs: cardiovascular diseases
DNMTs: DNA methyltransferases
ECM: extracellular matrix
H_2_O_2_: hydrogen peroxide
IPC: ischemic preconditioning
I/R: ischaemia-reperfusion injury
LVEF: LV ejection fraction
MI: myocardial infarction
MPs: myofibrillar proteins
NADPH: nicotinamide adenine dinucleotide phosphate
NO^-^: nitroxyl anion
NO_3_^-^: peroxynitrite
NO^+^: nitrosonium cation
NOX: nicotinamide adenine dinucleotide phosphate oxidase
O_2_^-^: superoxide anion
∙OH: hydroxyl radical
PCI: percutaneous coronary intervention
ROS: reactive oxygen species
SOD: superoxide dismutase
SR: sarcoplasmic reticulum
STEMI: ST-elevation myocardial infarction
TTS: takotsubo syndrome
TnI: troponin I
cTns: cardiac troponins
miRNA: microRNA
ncRNA: non-coding RNA
